# Geteilter Tumor des Ober- und Unterlides

**DOI:** 10.1007/s00347-021-01489-z

**Published:** 2021-09-21

**Authors:** P. J. Gaca, A. Doulis, P. A. Wawer Matos, M. Lewandowicz, A. C. Rokohl, L. M. Heindl

**Affiliations:** 1 Zentrum für Augenheilkunde, Medizinische Fakultät und Universitätsklinikum Köln, Universität zu Köln, Kerpener Str. 62, 50937 Köln, Deutschland; 2 Centrum für Integrierte Onkologie (CIO) Aachen-Bonn-Köln-Düsseldorf, Köln, Deutschland; 3 Abteilung für onkologische Chirurgie, Multidisziplinäres M. Copernicus Woiwodschaftszentrum für Onkologie und Traumatologie in Lodz, Lodz, Polen

## Anamnese

Eine 12-jährige Patientin stellte sich in unserer ophthalmoonkologischen Sprechstunde mit einer seit Geburt vorhandenen, pigmentierten und seit 1 Jahr in der Dicke progredienten, schmerzlosen Raumforderung am linken medialen Ober- und Unterlid vor. Die Augenanamnese war bis auf einen geringen myopen Astigmatismus leer. Anamnestisch bestanden keine Allgemeinerkrankungen, kein Trauma, keine B‑Symptomatik oder andere Beschwerden. Bis auf positive Allergieanamnese gegen Pollen war die Patientin komplett gesund. Die Patientin nahm keinerlei Medikamente ein. Auch die Familienanamnese bezüglich ophthalmologischer Erkrankungen war leer.

## Klinischer Befund

**Figure 1:**
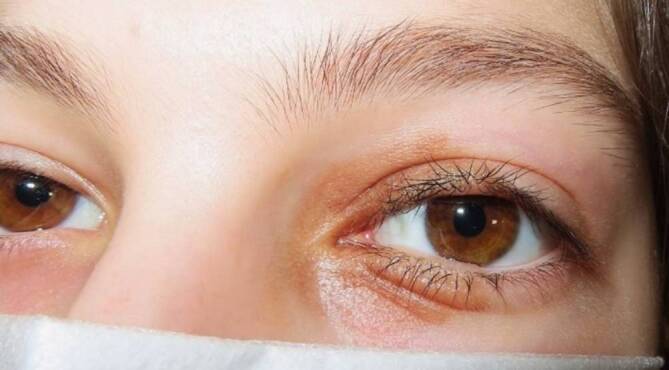

Die 12-jährige Patientin befand sich in einem sehr guten, altersüblichen Allgemein- und Ernährungszustand. Der Fernvisus lag beidseits mit bestmöglicher Refraktion bei 1,0. Die Motilität beider Augen war frei. In der weiteren klinischen ophthalmologischen Untersuchung zeigte sich am linken Auge eine schmerzlose, nicht druckdolente, scharf und regelmäßig begrenzte hellbraun pigmentierte, plakoide Raumforderung mit gering papillomatöser Oberfläche des medialen Ober- und Unterlides (Abb. 1). Der Tumor imponierte durch die Lidspalte gleichsam geteilt. Der restliche vordere Augenabschnitt war unauffällig. Die Untersuchung des Augenfundus zeigte keinen pathologischen Befund.

## Wie lautet Ihre Diagnose?

Die Diagnose lautet kongenitaler geteilter melanozytärer Nävus des Ober- und Unterlides – auch als „kissing nevus“, „split ocular nevus“ und „panda nevus“ bezeichnet.

## Diskussion

**Figure 2:**
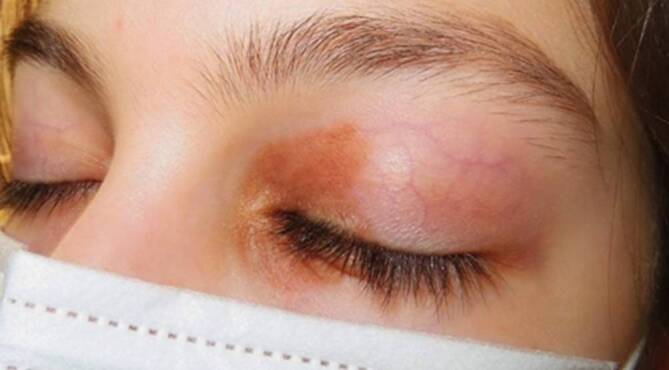

Geteilte Nävi des Ober- und Unterlides sind seltene melanozytäre Veränderungen der Augenlider. Sie wurden erstmals 1919 von Fuchs beschrieben [3]. In der Literatur wurden bisher etwa 120 solcher Fälle beschrieben [5]. Bei einem geteilten Nävus sind am oberen und unteren Lid gleichzeitig 2 getrennte Nävi vorhanden, die bei geschlossenem Auge eine Einheit bilden (Abb. 2). Geteilte Nävi sind in der Regel im medialen Bereich des Augenlides lokalisiert, obwohl sie auch in den medialen Lidwinkeln auftreten können. Die Augenlider sind bei Weitem die häufigste Lokalisation eines geteilten Nävus. Geteilte Nävi wurden auch an anderen Stellen des Körpers beschrieben. Der Penis und die Fingerzwischenräume sind weitere jedoch viel seltenere Lokalisationen gepaarter Läsionen [5].

Die Manifestation an gegenüberliegenden Arealen von Ober- und Unterlid ist Ausdruck der Embryonalentwicklung. Die Augenlider fusionieren in der 9. bis 10. Embryonalwoche (Woche post conceptionem), (38–40 mm, Scheitel-Steiß-Länge). Die Anlage des Nävus erfolgt im Stadium der fusionierten Lider [1, 4].

Als Abkömmlinge des Neuroektoderms wandern Melanozytenvorläufer während der Embryogenese als Melanoblasten aus der dorsalen Neuralleiste über die Dermis in die Epidermis in die verschiedenen Regionen des Embryos ein [1, 4]. Zwischen der 12. und 14. Embryonalwoche (100–120 mm) erfolgt die Wanderung zur Unterseite der embryonalen Epidermis der Kopfhaut und des Gesichts [4]. Aufgrund ihres Ursprungs in der Neuralleiste können Melanoblasten lokalisierte Proliferationen in den intradermalen, junktionalen und Compoundnävi zeigen [4].

**Figure 3:**
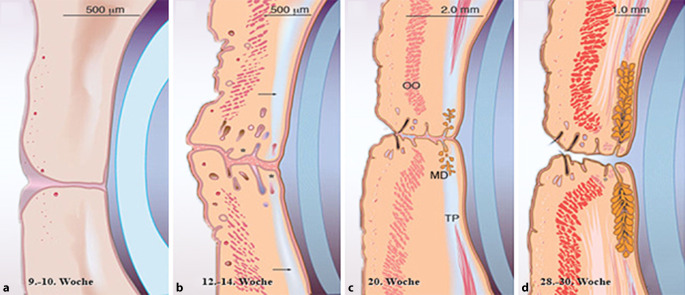

Während der 20. Embryonalwoche beginnen sich die Augenlider zu trennen, nachdem sich Lipide in der dermoepidermalen Junktionszone ansammeln. Durch die spätere Trennung von Ober- und Unterlid in der 28. bis 30. Embryonalwoche (180–200 mm) kommt es zur Teilung des Nävus [1]. Daraus kann man ableiten, dass Nävi in dieser Lokalisation spätestens zu diesem Zeitpunkt vorhanden sein müssen (Abb. 3). Interessanterweise wurde kein histologischer Unterschied in nävalen Subtypen (junktional, intradermal, compound) zwischen Ober- und Unterlid beschrieben [2].

Die Differenzialdiagnose des geteilten Nävus umfasst Papillome, Neurofibrome, Epitheliome. In der Vergangenheit waren es Tuberkulome und Syphilide [2].

**Diagnose:** Kongenitaler geteilter melanozytärer Nävus

Basierend auf dem Durchmesser (in cm), können die Nävi wie folgt eingeteilt werden:

a) klein (< 1,5 cm), b) mittel (1,5–19,9 cm), c) groß (> 20 cm).

Eine maligne Entartung bei kleinen oder mittleren Nävi wurde bisher nicht beobachtet [2]. Das Risiko einer malignen Transformation in ein malignes Melanom ist bei großen kongenitalen Nävi gut bekannt, bei kleinen und mittelgroßen Läsionen, die häufiger bei geteilten Nävi auftreten, ist es jedoch weniger klar [6]. Die tatsächliche Inzidenz der malignen Entartung in der Literatur ist sehr variabel und reicht von 2–40 % je nach Dauer der Nachbeobachtung mit einem Durchschnitt von 14 % für das gesamte Leben [6].

Kissing-Nävi der Augenlider müssen regelmäßig kontrolliert werden. Eine therapeutische Entfernung (Laser‑, Kryo, chirurgische Exzision mit ophthalmoplastischer Deckung) ist aufgrund des niedrigen Entartungsrisikos selten medizinisch indiziert. Sie kann bei sekundärer Amblyopie bei Ptosis, Epiphora oder Kompression der Puncta lacrimalia oder bei kosmetischem Wunsch erfolgen.

## Fazit für die Praxis

Zusammenfassend sind geteilte Nävi des Ober- und Unterlides seltene melanozytäre Veränderungen der Augenlider, die regelmäßig kontrolliert werden müssen. Eine therapeutische Entfernung (Laser‑, Kryo, chirurgische Exzision mit ophthalmoplastischer Deckung) ist aufgrund des niedrigen Entartungsrisikos selten medizinisch indiziert. Sie kann bei sekundärer Amblyopie bei Ptosis, Epiphora oder Kompression der Puncta lacrimalia oder bei kosmetischem Wunsch erfolgen.
